# Supplementary material for: Variations in Microstructural and Physicochemical Properties of Candelilla Wax/Rice Bran Oil–Derived Oleogels Using Sunflower Lecithin and Soya Lecithin
Source: Gels. 2021 Nov 22;7(4):226. doi: 10.3390/gels7040226 (PMC8628760; doi:10.3390/gels7040226)
Supplement: Supplementary file 1 [file gels-07-00226-s001.zip › gels-1327222-supplementary.pdf]

Variations in Microstructural and Physicochemical Properties of Candelilla Wax/Rice Bran Oil-Derived Oleogels Using Sunflower Lecithin and Soya Lecithin

Deblu Sahu [1], Deepti Bharti [1], Doman Kim [2], Preetam Sarkar [3] and Kunal Pal [1,*]

[1] Department of Biotechnology and Medical Engineering, National Institute of Technology, Rourkela 769008, Odisha, India
[2] Department of International Agricultural Technology & Institute of Green BioScience and Technology, Seoul National University, Seoul 151747, Gwangwon-do, Korea
[3] Department of Food Process Engineering, National Institute of Technology, Rourkela 769008, Odisha, India
* Correspondence: kpal.nitrkl@gmail.com

Supplementary materials

1. Polarized light microscopy

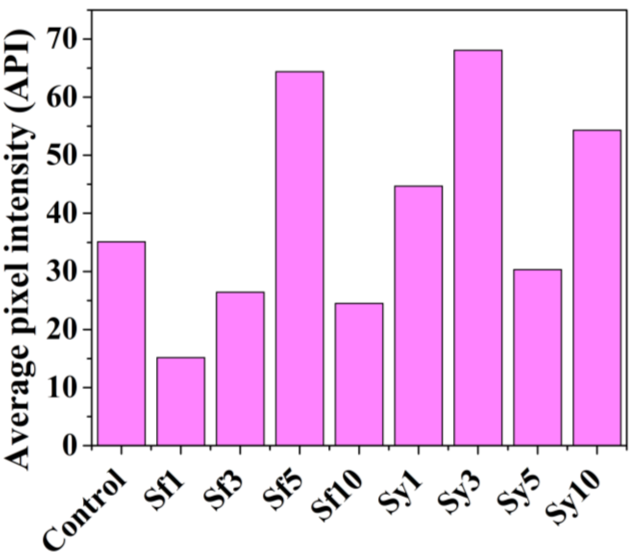

Figure S1. Average pixel intensity values obtained from polarized micrographs of the samples.

2. FTIRanalysis

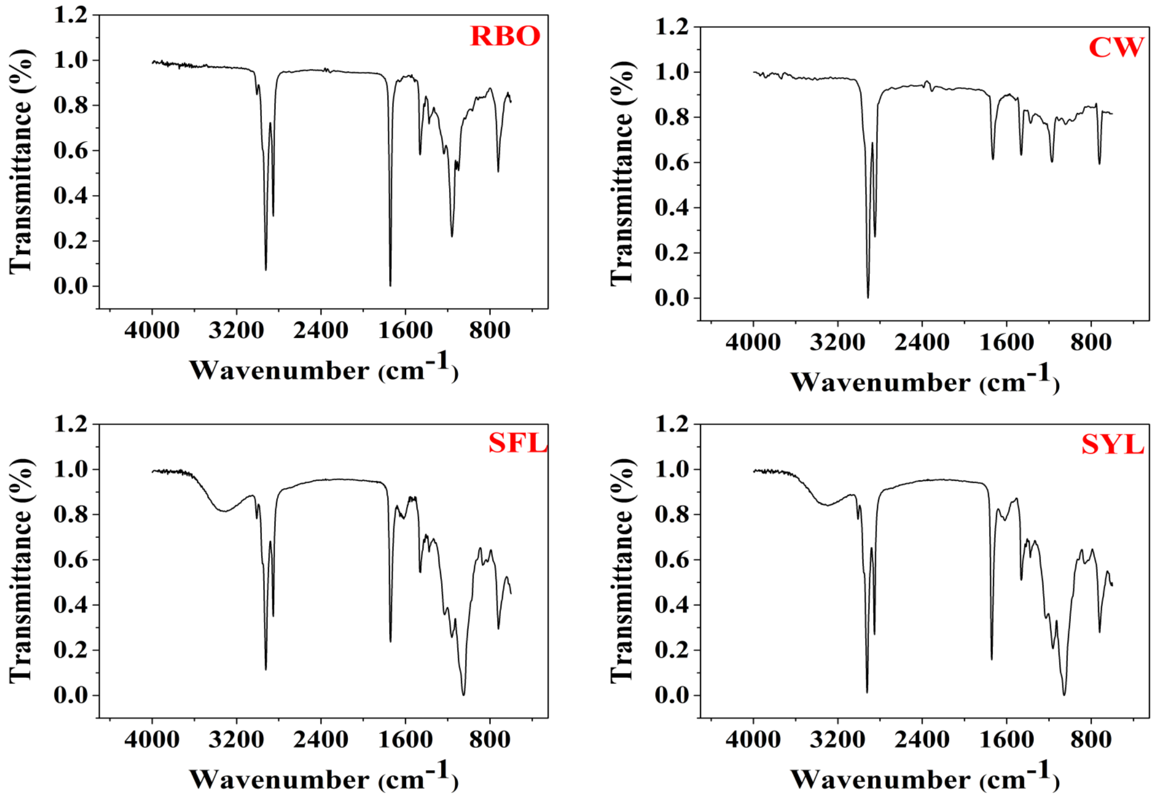

Figure S2. FTIR spectral profile of raw components.

3. Crystallization kinetics

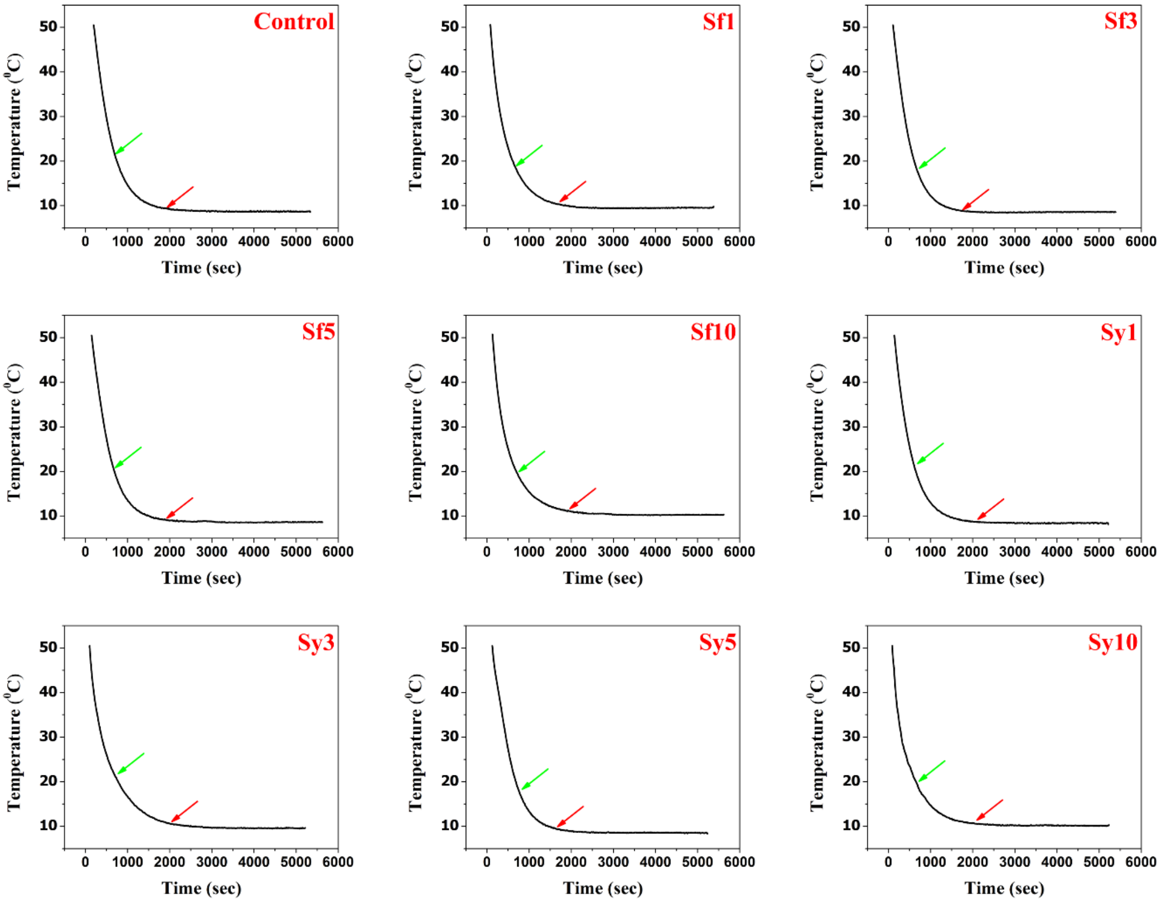

Figure S3. Gelation kinetics profiles of the prepared samples.

4. DSC analysis

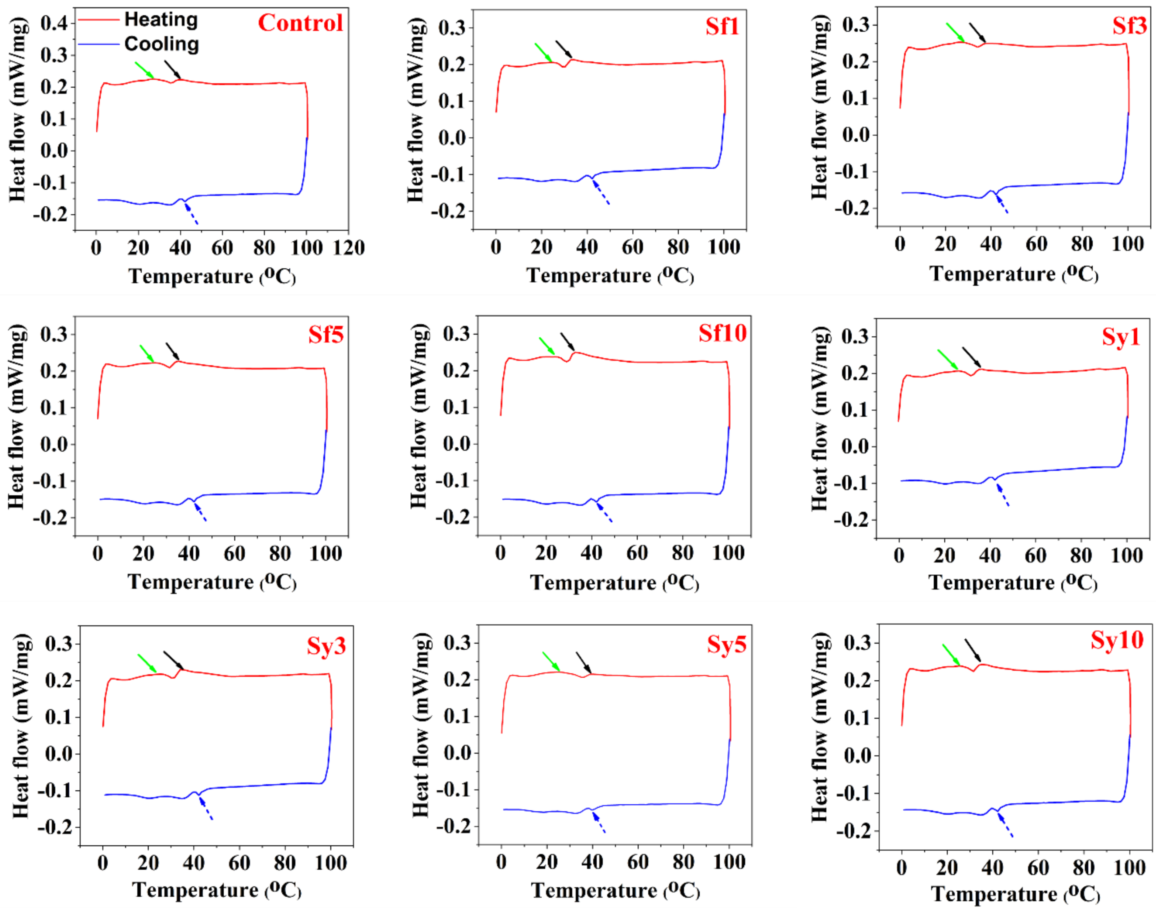

Figure S4. DSC melting and crystallization profiles of the prepared samples.

5. Determination of critical gelation concentration (CGC)

The minimum gelator concentration required to form oleogel is known as the CGC. The successful gel formation is confirmed through the non-occurrence of flow when the tubes are inverted. To predict the CGC, we started with the minimum, i.e., 1% (w/w) of CW for gelling RBO and moved till 5% (w/w) of CW. The required amount was allowed to melt in the oil at 90 °C for 25 min to dissolve the CW. The precise mixture was then cooled at room temperature for 5 minutes and kept under refrigeration for gelation. As seen in Figure S3(a-d), the amount of gelator used was insufficient to hold the oil, and therefore, the oil flows on tilting or

inverting. However, at 5% w/w of CW, the oleogel was formed successfully and did not flow on inverting the tube.

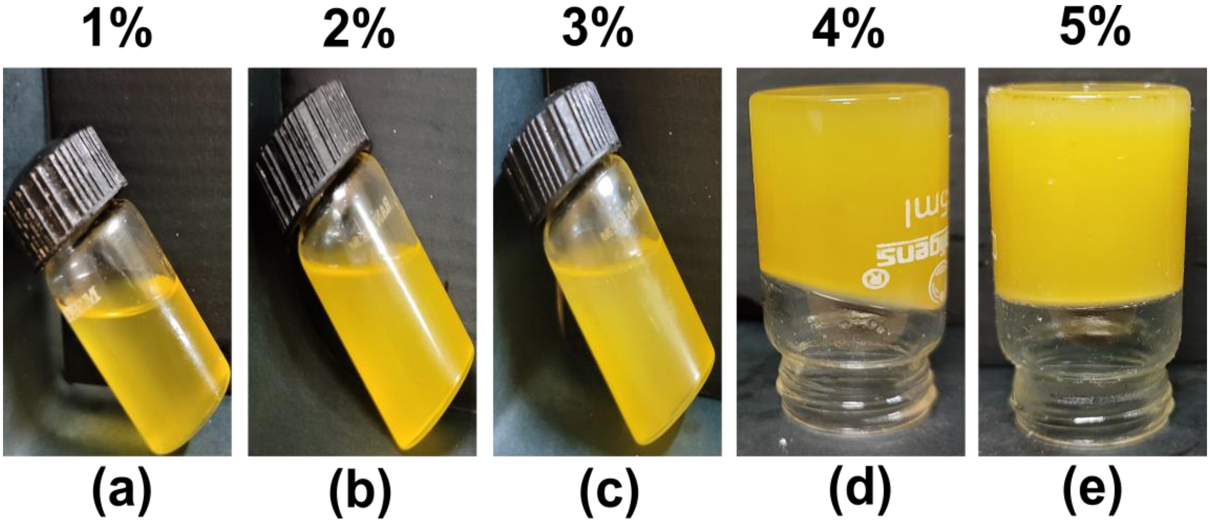

Figure S5. Confirmation of oleogel formation through inverted tube method.
